# Pre-diabetes is a predictor of short-term poor outcomes after acute ischemic stroke using IV thrombolysis

**DOI:** 10.1186/s12883-021-02102-1

**Published:** 2021-02-13

**Authors:** Byoung-Gwon Kim, Ga Yeon Kim, Jae-Kwan Cha

**Affiliations:** 1 Department of Preventive Medicine, College Of Medicine, Dong-A University, Busan, Korea; 2 Stroke Center, Department of Neurology, College of Medicine, Dong-A University, 1,3Ga, Dongdaeshin-Dong, Seo-Gu, Busan, 602-715 South Korea

**Keywords:** Stroke, Thrombolysis, Diabetes, Pre-diabetes

## Abstract

**Backgrounds:**

Pre-diabetes is an intermediate state between normal glucose metabolism and diabetes. Recent studies suggest that the presence of pre-diabetes is associated with poor outcomes after AIS. However, the results have been controversial. This study examines whether pre-diabetes influences the patients’ short and long-term outcomes for AIS using IV thrombolysis.

**Methods:**

We enrolled 661 AIS patients with IV thrombolysis. Based on the 2010 ADA guidelines, patients were classified as pre-diabetes, with HbA1c levels of 5.7–6.4%; diabetes, with HbA1c levels more than 6.5%; and NGM (normal glucose metabolism), with HbA1c levels less than 5.7%. We investigated short-term outcomes, including early neurologic deterioration (END), in-hospital death, and poor functional outcomes (mRS > 2) at 90 days. As for long-term outcomes, poor functional outcomes were measured at 1 year.

**Results:**

Of the 661 AIS patients treated with IV thrombolysis, 197 patients (29.8%) were diagnosed with pre-diabetes, and 210 (31.8%) were diagnosed with diabetes. In a multivariate analysis, pre-diabetes was an independent predictor for END (OR = 2.02; 95% CI 1.12–3.62; *p* = 0.02) and in-hospital death (OR = 3.12; 95% CI 1.06–9.09; *p* = 0.04). On the other hand, diabetes was a significant independent factor for poor long-term outcomes (OR = 1.75; 95% CI 1.09–2.78; *p* = 0.02) after correcting confounding factors.

**Conclusions:**

Unlike diabetes, pre-diabetes can be an important predictor of short-term outcomes after AIS. However, a more detailed research is needed to specify the precise mechanisms through which pre-diabetes affects the prognosis of acute ischemic stroke.

## Introduction

Diabetes is a well-known risk factor for stroke [1, 2] and a significant predictor for poor outcome after acute ischemic stroke [3–5]. Pre-diabetes, including impaired fasting glucose (IFG), impaired glucose tolerance (IGT) and/or impaired hemoglobin A1c (HbA1c), is an intermediate metabolic state between normal glucose metabolism and diabetes [6, 7].

Recent studies showed that pre-diabetes is associated with an increase of poor-outcome and mortality after acute ischemic stroke [8–10]. However, other reports did not observe a significant association between the presence of pre-diabetes and poor outcome after acute ischemic stroke after correcting several confounding factors [11, 12]. Also, only a few studies were able to identify a relationship between pre-diabetes and long-term outcome in acute ischemic stroke.

In this study, we investigated the impacts of pre-diabetes on short-term (early neurologic deterioration, in-hospital death, and functional outcome at 90 days) and long-term outcomes (functional outcome at 1-year) after treating acute ischemic stroke with IV thrombolysis. Also, we observed how pre-diabetes and diabetes differently influence outcomes in this population.

## Methods

### Study population

We performed a retrospective study approved by the Institutional Review Board in accordance with the ethical standards of the 1964 Declaration of Helsinki and its later amendments, incorporating consecutive patients enrolled in the database of Clinical Research Collaboration for Stroke in Korea (CRCS-K) registry [13].

We studied 3400 patients with AIS prospectively registered in the CRCS-K registry of Dong-A university stroke center from 2015 into August 2018. Among them, we reviewed 669 (19.7%) patients using IV t-PA. With eight patients excluded due to missing data, a total of 661 patients enrolled in this study.

### Data acquisition

The baseline data collected from the patients included the National Institutes of Health Stroke Scale (NIHSS) score. Stroke subtypes were determined by the consensus of two experienced neurologists who used a validated MRI-based algorithm [14] based on the Trial of Org 10,172 in Acute Stroke Treatment (TOAST) criteria [15].

Based on our stroke code system, we used intravenous tissue plasminogen activator (IV t-PA) within 4.5 h of stroke onset [16]. This study collected the onset-to-needle time (OTN, from symptom onset to IV t-PA use).

Based on the 2010 ADA guidelines [6, 7], all AIS patients with HbA1c levels within the range of 5.7–6.4% were classified as pre-diabetics, patients with HbA1c levels more than 6.4% were classified as diabetics, and patients with HbA1c levels less than 5.7% were classified as normal glucose metabolism (NGM). In this study, patients who were using antidiabetic drugs or had a history of diabetes were classified as diabetics regardless of their HbA1 levels. When the patients’ initial blood glucose level measured in the emergency room was above 140 mg/dl, it was defined as hyperglycemia [17].

In this study, we set early neurologic deterioration (END), in-hospital death, and poor functional outcome at 90 days as short-term outcomes and considered poor functional outcomes at 1 year as long-term outcomes. The definition of END [3] followed the criteria set by the CRCS-K registry as any new neurological symptoms/signs or neurological worsening that occurred within 3 weeks after the onset of index stroke. Aggravated neurological status includes 1) an increase in NIHSS score ≥ 2-point; 2) increase in NIHSS score 1a, 1b or 1c subscore ≥1-point; 3) increase in NIHSS score 5a, 5b, 6a, 6b (motor sub-score) ≥1-point; or 4) any kind of newly developed neurological symptom. The mRS scores were dichotomized into ≤2 (good functional outcome) and > 2 (poor outcome). Outcomes were measured by three trained nursing staff coordinators independent of the study. The mRS data were collected through phone calls every 3 month and 1-year after index stroke. Above stroke outcomes were prospectively collected in the registry using standardized protocols. The monthly meetings of the steering committee monitored the adequacy of case registration throughout the study.

### Statistical analysis

The clinical characteristics of the patients were summarized, and specific subgroups were described using descriptive statistics. After descriptive analyses, the categorical variables of the groups were compared using the Fisher’s exact test or Pearson’s chi-square test, while their continuous variables were compared using the Student’s t-test and ANOVA test. In this study, the initial glucose level (≥ 140 or < 140 mg/dl) and NIHSS (≥ 10 or < 10 points) were considered as categorical parameters.

Univariate analyses were performed to identify the odds ratio across the outcome parameters (END, in-hospital death, poor functional outcome at 90 days and 1-year). Also, logistic analyses were performed with clinical variables and laboratory results. Age, sex, baseline NIHSS, stroke subtype, atrial fibrillation, previous stroke, smoking, LDL cholesterol level, onset to needle time, mechanical thrombectomy, and baseline blood glucose level for END, in-hospital death, poor outcome at 90 days or poor outcome at 1-year were estimated by a logistic regression analysis [11, 18].

The odds ratios (ORs) and 95% confidence interval (CI) were calculated. All *P*-values were 2-sided, and statistical significance was defined as a P-value less than 0.05. STATA/MP 16.1 (StataCorp., College Station, TX, USA) was used in all the statistical analyses.

## Results

Among 661 AIS patients with IV thrombolysis, 197 patients (29.8%) were pre-diabetes, and 210 were diabetes (31.8%). Of the 210 patients with diabetes, 52 patients (7.9%) had unrecognized diabetes.

Table 1 compares the baseline characteristics and clinical outcomes in patients with normal glucose metabolism (NGM), pre-diabetes, and diabetes. The prevalence of hypertension and hyperlipidemia was significantly higher in patients with diabetes than those with NGM or pre-diabetes. Also, initial hyperglycemia (≥ 140 mg/dl) was much higher in the diabetes group than in others. However, there was no considerable difference in age or stroke severity, expressed by NIHSS, among the three groups. Also, their rates of mechanical thrombectomy and onset to needle time were similar.

**Table 1:** Baseline characteristics

	NGM (=254)	Pre-DM (=197)	DM (=210)	***p***
**Age**	66.9 ± (13.7)	68.7 ± 12.6	70.7 ± 10.0	0.27
**Female (%)**	99 (38.9)	82 (41.6)	82 (39.1)	
**Hypertension (%)**	135 (53.2)	110(55.8)	152 (72.4)	< 0.01
**Smoking (%)**	40 (15.8)	31 (15.7)	42 (20.0)	0.25
**Hyperlipidemia (%)**	42 (16.5)	39 (19.8)	86 (41.0)	< 0.01
**Prior CAD (%)**	29 (11.4)	31 (15.7)	39 (18.6)	0.09
**Atrial fibrillation (%)**	95 (37.4)	88 (44.7)	78 (37.1)	0.2
**Prior stroke (%)**	24 (9.5)	22 (11.2)	34 (16.2)	0.08
**TOAST classification**				0.48
Large artery atherosclerosis (%)	54 (21.3)	45 (22.8)	59 (28)	
Cardioembolism (%)	95 (37.4)	85 (43.2)	74 (35.2)	
Small vessel occlusion (%)	36 (14.2)	20 (10.2)	20 (9.5)	
Other (%)	5 (2.0)	3 (1.5)	3 (1.4)	
Undetermined (%)	64 (25.2)	44 (22.3)	54 (25.7)	
**NIHSS (median)**	9	10	9	
**NIHSS** ≥ **10 (%)**	129 (50.8)	117 (59.4)	114 (54.3)	0.91
**Initial glucose**	125.4 ± 25.7	133.9 ± 42.1	177.7 ± 70.6	< 0.01
**Initial glucose** ≥ **140 (%)**	60 (23.6)	56 ± 28.4	132 ± 62.9	< 0.01
**OTN (min, mean** ± **SD)**	153.9 ± 119.9	155.5 ± 122.7	178.4 ± 170.1	0.82
**Endovascular Tx (%)**	84 (33.1)	64 (32.5)	69 (32.9)	0.99
**END (%)**	24 (9.5)	33 (16.8)	38 (18.1)	0.02
**In hospital death (%)**	5 (2.0)	14 (7.1)	17 (8.1)	< 0.01
**poor outcome-90 days (%)**	97 (38.2)	87 (44.2)	119 (56.7)	< 0.01
**poor outcome − 1 year (%)**	91 (35.8)	87 (44.2)	119 (56.7)	< 0.01

*NGM* Normal glucose metabolism, *DM* Diabetes mellitus, *CAD* Coronary artery diseases, *TOAST* Trial of Org 10172 in Acute Stroke Treatment, *OTN* Onset to needle, *END* Early neurological deterioration

Regarding the short-term outcomes after acute ischemic stroke using IV thrombolysis, 126 patients (14.5%) experienced an END, 36 patients (5.4%) an in-hospital death, and 303 (45.8%) a poor outcome at 90 days. One-year after using IV thrombolysis, 297 patients (44.9%) showed a poor functional outcome measured by mRS (Table 1). The prevalence of END (*p* < 0.05), in-hospital death (*p* < 0.05), poor-outcome at 90 days (*p* < 0.01), and 1-year (p < 0.01) were significantly higher in patients with diabetes than in those with NGM. In the pre-diabetes population, the proportion of END (*p* < 0.05) and in-hospital death (*p* < 0.01) were significantly higher compared to that of the NGM population.

In a univariate analysis, pre-diabetes (*p* = 0.02) and diabetes (*p* < 0.01) were significantly associated with END. Regarding in-hospital death, it was significantly related to pre-diabetes (*p* < 0.01), diabetes (*p* < 0.01), age (*p* < 0.01), female sex (*p* < 0.01), initial stroke severity (*p* < 0.01), and initial glucose level (*p* < 0.01). As for poor outcome at 90 days, diabetes (*p* < 0.01), age (*p* < 0.01), female sex (*p* < 0.01), hypertension (*p* < 0.01), previous history of coronary artery disease (*p* < 0.01), Atrial fibrillation (*p* < 0.01), initial stroke severity (*p* < 0.01), and initial glucose level (*p* < 0.01) had a significantly higher odd for it. In the case of poor outcome at 1-year, it was severely influenced by diabetes (*p* < 0.01), age (*p* < 0.01), female sex(*p* < 0.01), hypertension (*p* < 0.01), atrial fibrillation (*p* < 0.01), initial stroke severity (*p* < 0.01), and initial glucose level (*p* < 0.01).

In a multivariate analysis that controlled for relevant factors, the presence of pre-diabetes had an independent significance on the occurrence of END (OR = 2.02; 95% CI 1.12–3.62; *p* = 0.02) and in-hospital death (OR = 3.12; 95% CI 1.06–9.09; *p* = 0.04). In contrast, the presence of diabetes was a significant predictor for poor outcome at 1-year (OR = 1.75; 95% CI 1.09–2.78; p = 0.02) after correcting relating covariant. In this study, the presence of pre-diabetes predicted short-term outcomes such as END and in-hospital death. By comparison, diabetes was significant in predicting long-term outcome at 1-year after using IV thrombolysis in AIS. Initial glucose level more than 140 mg/dl (OR = 2.54; 95% CI 1.15–5.59; *p* < 0.01) was a significant predictor for in-hospital mortality (Table 2).

**Table 2:** Univariate and multivariate analysis for short- and long-term outcomes after AIS Using IV thrombolysis

	Univariate (95% CI)	OR	Multivariate (95% CI)	Adjusted OR
END				
Pre-diabetes	1.20–3.38	1.92	1.12–3.62	2.02
Diabetes	1.22–3.66	2.11	0.83–2.92	2.02
Initial hyperglycemia	0.98–2.37	1.53	0.83–2.29	1.38
In-hospital death				
Pre-diabetes	1.34–10.76	3.8	1.06–9.09	3.12
Diabetes	1.59–12.10	4.38	0.83–7.60	2.52
Initial hyperglycemia	1.75–7.29	3.59	1.15–5.59	2.54
Poor outcome at 90 days				
Pre-diabetes	0.88–1.86	1.58	0.63–1.51	0.98
Diabetes	1.45–3.07	2.11	0.97–2.45	1.54
Initial hyperglycemia	1.30–2.45	1.79	0.91–2.00	1.35
Poor outcome at 1-year				
Pre-diabetes	0.97–2.07	1.41	0.73–1.75	1.13
Diabetes	1.61–3.40	2.34	1.09–2.78	1.75
Initial hyperglycemia	1.28–2.41	1.76	0.86–1.89	1.27

Adjusted for Age, sex, baseline NIHSS, stroke subtype, atrial fibrillation, previous stroke, smoking, LDL cholesterol level, onset to needle time, mechanical thrombectomy, and baseline blood glucose level

*CI* Confidential interval, *END* Early neurologic deterioration, initial hyperglycemia- more than 140 mg/dl

## Discussion

The prevalence of diabetes in acute ischemic stroke was 31.8% in this study, similar to several other prospective stroke registry data [3, 19, 20]. Also, among the patients diagnosed with diabetes, nearly 25% were so-called unrecognized diabetes, newly diagnosed using HbA1C. Therefore, it has been recommended to check HbA1C or oral glucose tolerance test (OGTT) in all acute ischemic stroke patients, regardless of their previous history [21]. Among the recommended tests, HbA1C has an advantage over OGTT in identifying pre-diabetes in patients with acute stroke unaffected by the acute-phase reaction [6]. Using HbA1C, the prevalence of pre-diabetes was 29.8% in our study, similar to previous reports. In this study, the presence of diabetes had a predictable value for the long-term outcome at 1-year but not for short-term outcomes.

Several previous results demonstrated that the presence of diabetes was significantly associated with END and worse outcomes at 90 days after acute ischemic stroke [5, 22]. However, in the Bichat stroke registry [4], the presence of diabetes had no impact on END and functional outcomes at 90 days in AIS using IV thrombolysis. Also, in the Lausanne Stroke Registry [23], there was no difference in short-term functional outcomes between stroke patients with and without diabetes. Differences across the patient population, functional outcome scales and follow-up duration may account for the contradictory results.

In particular, after acute stroke, short-term outcomes are primarily influenced by the patient’s age and severity of stroke [24]. In our study, there were no differences in age and stroke severity among three groups, so it was unlikely that the presence of diabetes would affect prognosis from the beginning of the stroke. In contrast, cardiovascular risk factors such as diabetes significantly influenced outcomes at least 90 days after AIS. This late-appearing pattern is probably related to the progression of the atherosclerotic vascular disease over time, which is known to be more rapid among diabetes. Also, the vascular risk profile is worse among patients with diabetes because of their higher number of comorbidities and chronic conditions that lead to accelerated atherosclerosis [25, 26]. In the Danish national stroke registry, diabetes did not affect mortality in patients with first ischemic stroke until 30 days; however, it began to affect it after 90 days and increased it significantly in 1 year [25]. Therefore, it is reasonable to conclude that diabetes is more likely to affect the mid-to-long-term prognosis of acute stroke patients than their short-term outcomes.

Many previous studies showed that initial transient hyperglycemia higher than 140 mg/dl is an important predictor for in-hospital death after acute ischemic stroke using IV thrombolysis [2, 27, 28]. Our results also reaffirmed this notion. Data regarding the impact of transitory hyperglycemia on stroke mortality for more extended periods of follow-up are contradictory. Ninety days after stroke onset, the mortality rate became almost equal between patients with transitory hyperglycemia and diabetes. Their rates did not differ after 180 or 365 days. Furthermore, upon admission, transient hyperglycemia was not an independent risk factor for one-year mortality after stroke [2, 28]. These reports indirectly support the authors’ finding that transient hyperglycemia only affects short-term prognosis after acute ischemic stroke.

In this study, pre-diabetes had a significant impact on the occurrence of END and in-hospital death after AIS using IV thrombolysis. Although a few previous studies suggested an association between the presence of pre-diabetes after acute stroke and worse outcomes [8–10], their relationship remains controversial [11, 12].

Generally, patients with pre-diabetes have an increased risk of type 2 diabetes and higher risks of cardiovascular diseases, such as stroke and recurrent stroke. Pre-diabetes, an intermediate metabolic state between normal glucose metabolism and diabetes, indicates an increased risk of developing type 2 diabetes and cardiovascular diseases in the future. Therefore, pre-diabetes should not be considered as a distinct clinical entity. Instead, it should be regarded as a continuum of increasing levels that represent growing risks of developing diabetes [6]. Therefore, it is not logical to suggest that pre-diabetes and diabetes affect acute stroke in different directions.

In this study, AIS patients with pre-diabetes had a significantly lower prevalence of statin usage (19.3% vs. 30.0%, data are not shown) before AIS than those with diabetes. Also, the rate of previous use of antiplatelet agents was lower in the pre-diabetes population than in the diabetes population. These data suggest that the pre-diabetes patients in our study may have been alienated from appropriate medical measures despite their risk of stroke occurrence. For this reason, they could have suffered more severe damages in the acute phase after stroke. Also, the higher hypercoagulable state in pre-diabetes patients, absence of any interventions, might have been critical to their early vascular instability [29, 30] consequently leading to END after AIS using IV thrombolysis. However, unlike for diabetic patients, treatments that use antiplatelet drugs immediately after acute stroke or high doses of statins can ensure the stability of prediabetic patients’ vascular condition while having little effect on their long-term prognosis.

Since only IV thrombolytic patients among the AIS patients have been screened, this study has the advantage of reducing biases such as the onset time of stroke or treatment options that may affect long-term prognosis. Also, this study has several methodological strengths including prospective data collection, and predefined protocols that capture clinical outcomes after stroke. This study used a meticulous criterion of END that has been adopted by the prospective CRCS-K registry from 2009. Particularly, this END definition, unlike those used in other studies [4, 11, 22] is characterized by more detailed items and a longer observation period. Our recent study proved that even minor neurologic deteriorations during hospitalization due to index stroke and within 3 weeks of onset are associated with long-term poor outcomes after stroke [31].

In this study, we proved that the presence of diabetes is associated with a worse long-term outcome after acute ischemic stroke. Also, we suggested that the presence of pre-diabetes may have an impact on the short-term outcome. A plausible reason may be the lack of interest in identifying pre-diabetes in the pre-stroke stage. However, there is limitation in the confirmation above notion because this study did not have complete data on the patients’ use of drugs such as statin or health care before an index stroke. In a future research, a systematic review of the short-term and long-term prognosis for pre-diabetes in patients with acute ischemic stroke that uses a meticulous large-scale acute stroke registry will be needed.

## Data Availability

The datasets generated and/or analyzed during the current study are not publicly available due for they are personal data but are available from the corresponding author on reasonable request.
